# Urothelial Cell Carcinoma of the Renal Pelvis Misdiagnosed as Ureteropelvic Junction Obstruction: A Case Report

**DOI:** 10.3390/medicina57111158

**Published:** 2021-10-25

**Authors:** Jeong-Hyouk Choi, Tae-Soo Choi, Dong-Gi Lee, Gyeong-Eun Min

**Affiliations:** Department of Urology, College of Medicine, Kyung Hee University, Seoul 05278, Korea; jhchoi0@gmail.com (J.-H.C.); taesoochoi85@hanmail.net (T.-S.C.); urology@khu.ac.kr (D.-G.L.)

**Keywords:** carcinoma, transitional cell, ureteropelvic junction obstruction, pyeloplasty

## Abstract

Urothelial tumors are typically a disease affecting elderly individuals and are rare in young patients. Moreover, upper urinary tract urothelial carcinoma is extremely rare in the young age group. In this study, we present a case of urothelial cell carcinoma of the renal pelvis and ureter in a young man without risk factors of urothelial carcinoma, which was misdiagnosed as ureteropelvic junction obstruction and treated with a laparoscopic pyeloplasty.

## 1. Introduction

The mucosal surface of the renal pelvis, ureter, urinary bladder, and urethra is called the urothelium. The incidence of urothelial carcinoma (UC) is steadily rising worldwide. For example, bladder cancer is the most frequently occurring urinary tract malignancy and is also the 10th most common cancer in the world [1]. However, upper urinary tract urothelial carcinoma (UUT-UC) is relatively uncommon and accounts for approximately 5% of urothelial carcinoma (UC) cases [2]. UC is typically a disease affecting elderly individuals, with a male predominance, and is rare in young patients [3]. In this study, we present a case of UC of the renal pelvis and ureter in a young man, which was misdiagnosed as ureteropelvic junction (UPJ) obstruction (UPJO) and treated with a laparoscopic pyeloplasty. This case report is developed according to the CARE checklist (Supplemental Table S1).

## 2. Case Report

**Figure 1:**
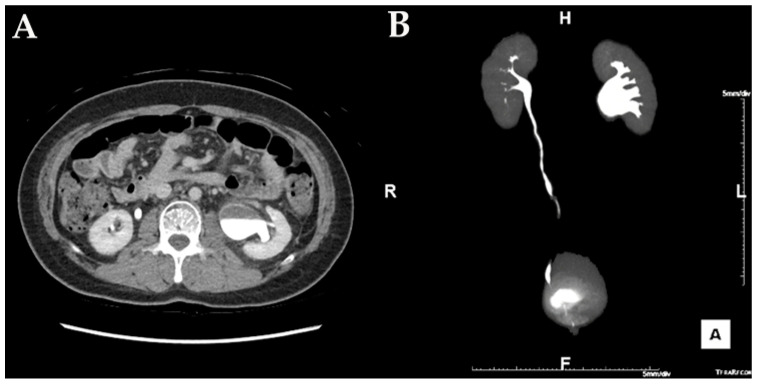
In the abdominal CT, hydronephrosis with a UPJ obstruction on left urinary tract is noted. (**A**) axial image of the left UPJO, (**B**) reconstructed coronal image of the left UPJO.

**Figure 2:**
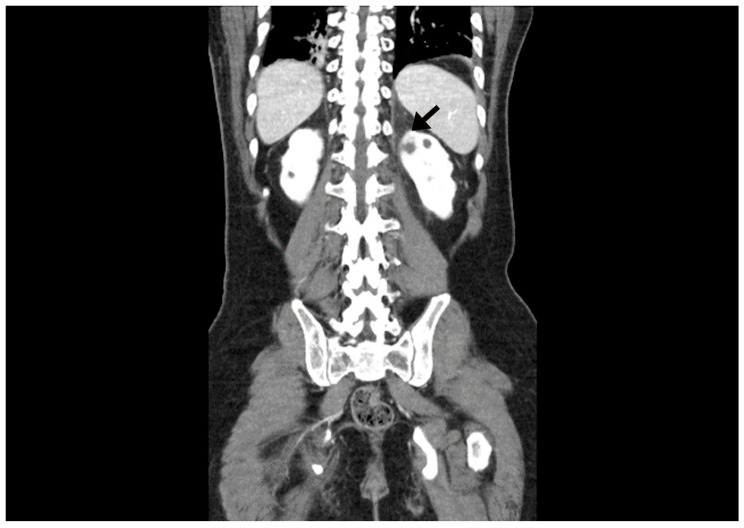
In the abdominal CT, an ill-defined low-density lesion (arrow) at the upper pole of the left kidney is noted.

**Figure 3:**
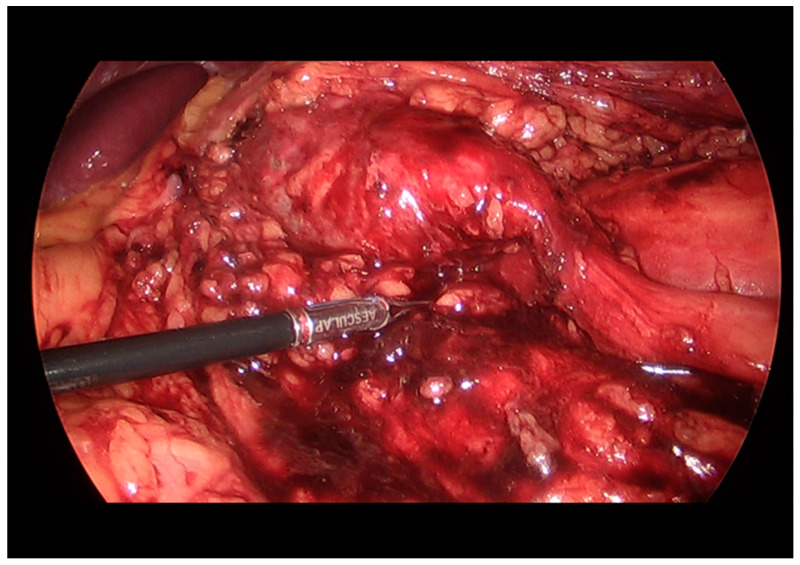
In the laparoscopy, a dilated left renal pelvis and abrupt narrowing of the UPJ is observed.

A 34-year-old man with cerebral palsy presented with high fever (39.5 °C), left flank pain for 3 days, and gross hematuria. Twenty years prior, he underwent a ureteroscopic stone removal and ureteral dilation at another hospital and had intermittent episodes of left renal colic and urinary tract infections. Urinalysis revealed the presence of hematuria (red blood cells: 30–50/HPF), pyuria (white blood cells: >50/HPF), and bacteriuria (bacteria: a few colonies/HPF). The C-reactive protein level was 10.3 mg/dL. An abdominal computed tomography (CT) revealed hydronephrosis with a narrowing of the UPJ (Figure 1). A low-density lesion of approximately 1.6 cm with subtle irregular margins in the upper pole of the left kidney, suspected to be a renal abscess, was observed (Figure 2). Urine cytology examinations were performed three times, but all were negative. After 3 days of parenteral antibiotic treatment, the patient became afebrile. The follow-up urinalysis results were normal. A follow-up CT, performed 3 months later, showed that the ill-defined low-density lesion at the upper pole of the left kidney had disappeared. hydronephrosis with a proximal ureteral narrowing was suggestive of ureteral UPJO associated with an improved state of combined ureteritis and a renal abscess. Suspecting recurrent left pyelonephritis due to the UPJO, laparoscopic pyeloplasty was planned. Prior to the pyeloplasty, the patient underwent retrograde pyelography (RGP). The RGP revealed a dilated left renal pelvis and the abrupt narrowing of the UPJ, and there was no filling defect in the left urinary tract. The surgery was performed using a transperitoneal approach. The laparoscopy revealed a UPJO (Figure 3). The dismembered technique was used for the reconstruction of the UPJ. The surgery was completed without complications. A pathological examination revealed that the excised stenotic segment had a UC with an invasion beyond the muscularis propria into the periureteral fat. This pathological stage was pT3, and the grade was high (World Health Organization (WHO) grade 3). Due to the possibility of residual cancer in the remaining ureter, we decided to perform a laparoscopic radical nephroureterectomy with a bladder cuff excision. Two weeks after the laparoscopic pyeloplasty, the patient underwent a second surgery. A pathological examination revealed residual tumor cells in the proximal ureter. The tumor invaded the subepithelial connective tissue (rpT1). The final pathological stage was pT3 with a high grade (WHO grade 3). There was a multifocal urothelial carcinoma in situ in the renal pelvis and mid-ureter. Postoperatively, the patient received four cycles of adjuvant chemotherapy with gemcitabine and cisplatin. The patient has been undergoing regular follow-up sessions without evidence of a recurrence or distant metastasis for 2 years.

## 3. Discussion

A UPJO is a common urological problem that may result in hydronephrosis and the deterioration of renal function. Although most commonly observed in pediatric populations, a UPJO in adults may arise from a number of etiologies, including congenital causes, and acquired stenosis due to urolithiasis; urothelial malignancy; and other retroperitoneal disease processes [4].

UC is the fourth most common cancer worldwide. Bladder cancer, the most common UC, accounts for 90% to 95% of cases, while UUT-UC, which is more malignant, accounts for only 5% to 10% of cases [3]. Approximately two thirds of patients who present with UUT-UCs have an invasive disease at the time of diagnosis, compared to 15–25% of patients presenting with muscle-invasive bladder tumors [5]. UUT-UCs have a peak incidence in individuals aged 70–90 years and are three times more common in men [6]. Urothelial tumors are rare in young patients [3]. If hydronephrosis is present in the abdominal CT of a patient at a young age, it is often presumed to be due to a benign disease, such as UPJO or an extrarenal pelvis rather than UUT-UCs. In addition, if a patient’s chief complaint is not gross hematuria, the possibility of UUT-UCs may be overlooked. The exposure to smoking and aristolochic acid plays a causative role in the development of UUT-UCs [7,8]. The exposure to cigarette smoke increases the relative risk of developing UUT-UCs from 2.5 to 7.0 [9,10]. In some countries, the presence of arsenic in drinking water has been tentatively linked to upper tract urothelial carcinomas [11,12,13].

Our patient exhibited mental retardation and cerebral palsy since early childhood; therefore, it was presumed that the possibility of exposure to tobacco or occupational chemicals, which are major environmental risk factors for UC, was low. The patient had a history of urolithiasis from adolescence, and he underwent a ureteroscopic stone removal after ureteral dilation, due to a ureteral stricture, although no kidney stones were found in the abdominal CT at the time of the diagnosis of UCs. A few studies have concluded that urolithiasis may increase the risk of UCs, suggesting that continuous stimulation by the presence of stones and the obstruction to the urothelium affects tumor formation [8,14,15].

Case reports have described UPJO being misdiagnosed as UC, but the patients were a chronic smoker older than 40 years of age [16] and a 70-year-old patient with gross hematuria [4]. Nomikos et al. studied 31 patients with bladder UC who were younger than 40 years of age. Amongst them, 87% were tobacco smokers, and 13% had a family history of bladder cancer. A total of 9.6% had occupational exposure to the risk factors. A total of 90% of the patients had non-muscle-invasive bladder cancer (NMIBC), and only 10% had invasive disease [17]. Wen et al. also reviewed 30 patients younger than 40 years of age. Eighty percent of these patients had NMIBC [18]. Based on these studies, when young adults are diagnosed with bladder cancer, they are more likely to have low-stage tumors with progression similar to that seen in elderly individuals. However, unlike in cases of bladder cancer, 60% of patients have invasive cancer at the time of UUT-UC diagnosis [3]. Therefore, even if a young patient does not show clinical features suggestive of cancer, such as gross hematuria or a positive result on urine cytology, and imaging studies show obstructions with an enhancement along the urinary tract, a thorough evaluation should be performed for the early detection of invasive cancer and appropriate methods of intervention for its treatment should be employed.

## 4. Conclusions

Even in young adults with a UPJO, as with elderly patients, aggressive methods of evaluation and treatment are required if UUT-UC is suspected based on imaging studies.

## Data Availability

Data sharing not applicable.

## Supplementary Materials

The following materials are available online at https://www.mdpi.com/article/10.3390/medicina57111158/s1, Supplementary Table S1: CARE Checklist (2013) of information to include when writing a case report.
