# Supplementary material for: Urothelial Cell Carcinoma of the Renal Pelvis Misdiagnosed as Ureteropelvic Junction Obstruction: A Case Report
Source: Medicina (Kaunas). 2021 Oct 25;57(11):1158. doi: 10.3390/medicina57111158 (PMC8622892; doi:10.3390/medicina57111158)
Supplement: Supplementary file 1 [file medicina-57-01158-s001.zip › medicina-1402338-SI.pdf]

Topic	Item	Checklist item description	Reported on page
**Title**	**1**	The words “case report” should be in the title along with what is of greatest interest in this case	Page 1
**Key Words**	**2**	The key elements of this case in 2 to 5 key words	Page 1
**Abstract**	**3a**	Introduction—What is unique about this case? What does it add to the medical literature?	Page 1
	**3b**	The main symptoms of the patient and the important clinical findings	Page 1
	**3c**	The main diagnoses, therapeutics interventions, and outcomes	Page 1
	**3d**	Conclusion—What are the main “take-away” lessons from this case?	Page 1
**Introduction**	**4**	Brief background summary of this case referencing the relevant medical literature	Page 1
**Patient Information**	**5a**	Demographic information (such as age, gender, ethnicity, occupation)	Page 1
	**5b**	Main symptoms of the patient (his or her chief complaints)	Page 1
	**5c**	Medical, family, and psychosocial history including co-morbidities, and relevant genetic information	Page 1
	**5d**	Relevant past interventions and their outcomes	Page 1
**Clinical Findings**	**6**	Describe the relevant physical examination (PE) findings	
**Timeline**	**7**	Depict important milestones related to your diagnoses and interventions (table or figure)	
**Diagnostic Assessment**	**8a**	Diagnostic methods (such as PE, laboratory testing, imaging, questionnaires)	Page 1-2
	**8b**	Diagnostic challenges (such as financial, language, or cultural)	
	**8c**	Diagnostic reasoning including other diagnoses considered	Page 1-2
	**8d**	Prognostic characteristics (such as staging in oncology) where applicable	Page 2
**Therapeutic Intervention**	**9a**	Types of intervention (such as pharmacologic, surgical, preventive, self-care)	Page 1-2
	**9b**	Administration of intervention (such as dosage, strength, duration)	
	**9c**	Changes in intervention (with rationale)	Page 2
**Follow-up and Outcomes**	**10a**	Clinician-assessed outcomes and when appropriate patient-assessed outcomes	Page 2
	**10b**	Important follow-up test results	Page 2
	**10c**	Intervention adherence and tolerability (How was this assessed?)	
	**10d**	Adverse and unanticipated events	Page 1-2
**Discussion**	**11a**	Discussion of the strengths and limitations in the management of this case	Page 3-4
	**11b**	Discussion of the relevant medical literature	Page 3-4
	**11c**	The rationale for conclusions (including assessment of possible causes)	Page 3-4
	**11d**	The main “take-away” lessons of this case report	Page 4
**Patient Perspective**	**12**	Did the patient share his or her perspective or experience? (Include when appropriate)	Page 2
**Informed Consent**	**13**	Did the patient give informed consent? Please provide if requested	**Yes _V_ No ___**

Table S1. CARE Checklist (2013) of information to include when writing a case report.
